# Supplementary material for: Identification and functional analysis of non-coding regulatory small RNA FenSr3 in Bacillus amyloliquefaciens LPB-18
Source: PeerJ. 2023 May 15;11:e15236. doi: 10.7717/peerj.15236 (PMC10194069; doi:10.7717/peerj.15236)
Supplement: Supplemental Information 4 [file peerj-11-15236-s004.zip › KO/CK-vs-T1_map/map04714.html]

KEGG PATHWAY: Thermogenesis - Reference pathway


|  |  |
| --- | --- |
| **Thermogenesis - Reference pathway** |  |

[
Pathway menu
| Organism menu
| Pathway entry
| Show description
| User data mapping
]

Thermogenesis is essential for warm-blooded animals, ensuring normal cellular and physiological function under conditions of environmental challenge. Thermogenesis in brown and beige adipose tissue is mainly controlled by norepinephrine, which is released from sympathetic nervous system in response to cold or dietary stimuli. The mitochondrial uncoupling protein 1 (UCP1) is responsible for the process whereby chemical energy is converted into heat in these adipocytes. Activation of these adipocytes leads to an increase in calorie consumption and is expected to improve overweight conditions, providing a potential strategy for treating obesity and its related metabolic disorders.

|  |  |  |
| --- | --- | --- |
| Reference pathway | 184% 150% 122% 100% 82% 67% 55% | 图片下载 |
